# Supplementary material for: Morphology Effect of Bismuth Vanadate on Electrochemical Sensing for the Detection of Paracetamol
Source: Nanomaterials (Basel). 2022 Apr 1;12(7):1173. doi: 10.3390/nano12071173 (PMC9000780; doi:10.3390/nano12071173)
Supplement: Supplementary file 1 [file nanomaterials-12-01173-s001.zip › nanomaterials-1634265-supplementary.pdf]

Supporting Information

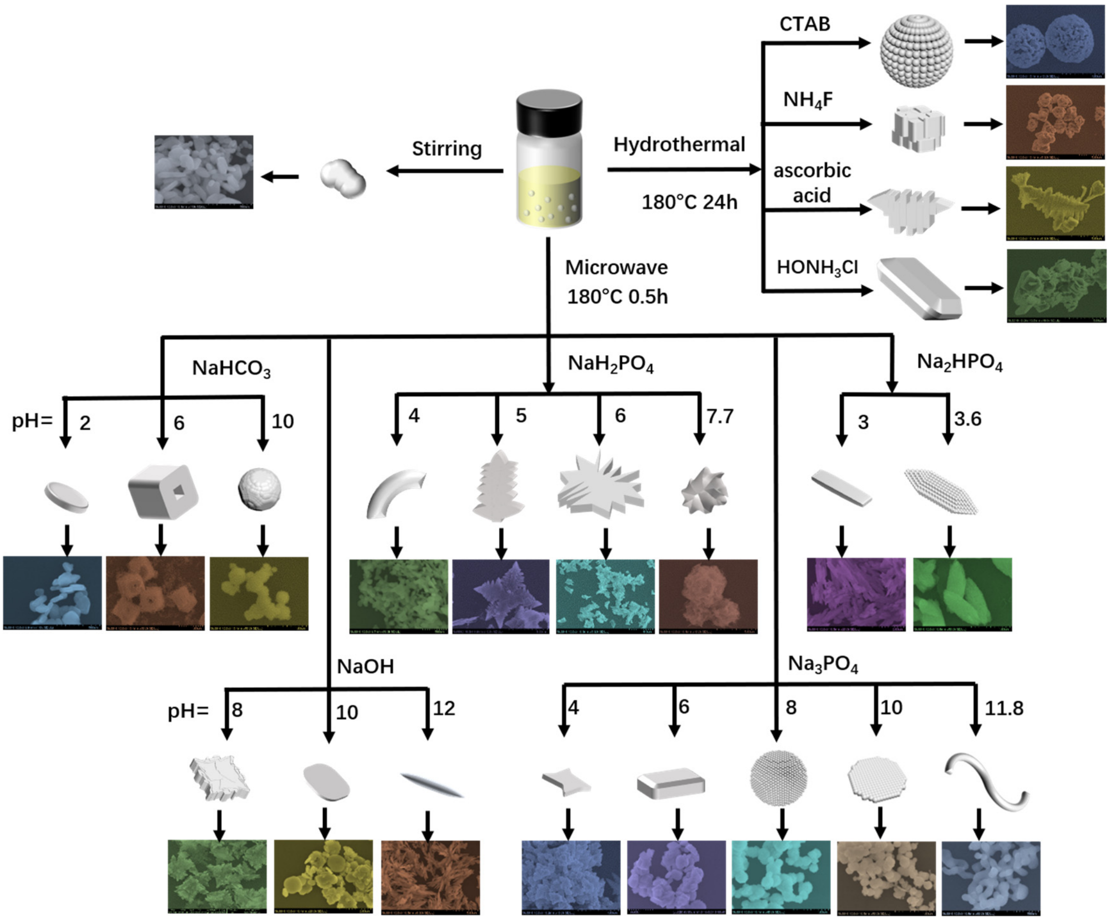

Figure S1. The synthesis produces of morphology-dependent $BiVO_4$ by stirring, hydrothermal and micro wave methods.

The $BiVO_4$ powders were synthesis by stirring, hydrothermal and micro wave methods, respectively. As a control sample, the nanoparticle $BiVO_4$ was prepared by stirring 30 min with the mixture of $Bi(NO_3)_3$ and NH_4VO_4. Beside, the hydrothermal approach also used as a reference experimental method. The hexadecyl trimethyl ammonium bromide (CTAB, Sigma-Aldrich, ⩾99%), NH_4F (Sigma-Aldrich, ⩾99.99%), L-ascorbic acid (TCI, >99.0%) and $HONH_3Cl$ (Skychy Group, AR) were used as surfactant

to obtain the different morphology of $BiVO_4$ (sphere, cube, irregular and cuboid shapes). As a short time and efficient preparation method, the microwave approach can shorten the preparation time effectively, from 24 hours to 30 minutes. The morphology of $BiVO_4$ can be controlled with different additives, including NaH_2PO_4, Na_2HPO_4, Na_3PO_4, Na_2CO_3 and NaOH. The pH values of solution from 2 to 10 were further adjustd by adding HCl or NaOH. Due to the difference in ionization and hydrolysis of phosphate, carbonate and alkaline, the morphology of bismuth vanadate could form many different shapes, including nanosheet, tetrahedron, cuboid, sphere and irregular shapes.

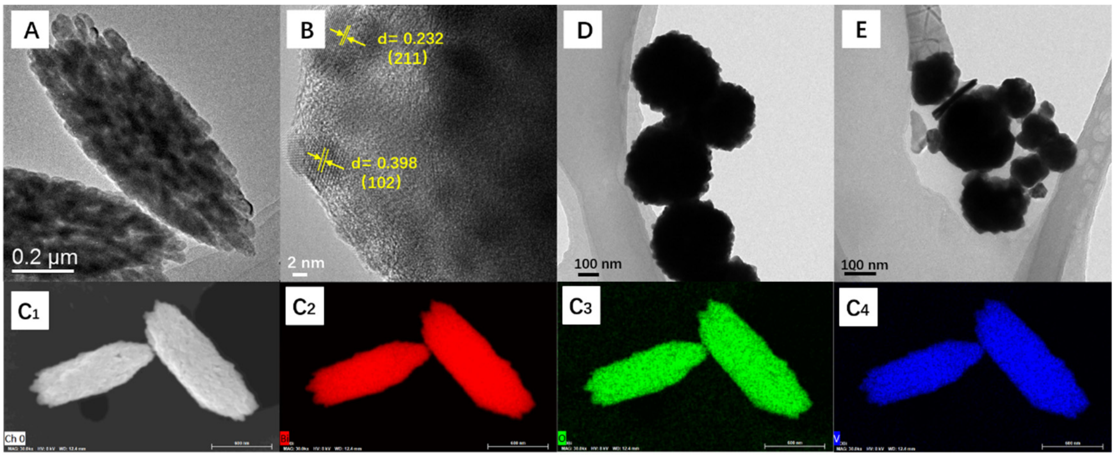

Figure S2. (A) TEM, (B) HRTEM and EDS mapping images of fusiform $BiVO_4$. TEM images of (D) flowered and (E) particle $BiVO_4$.

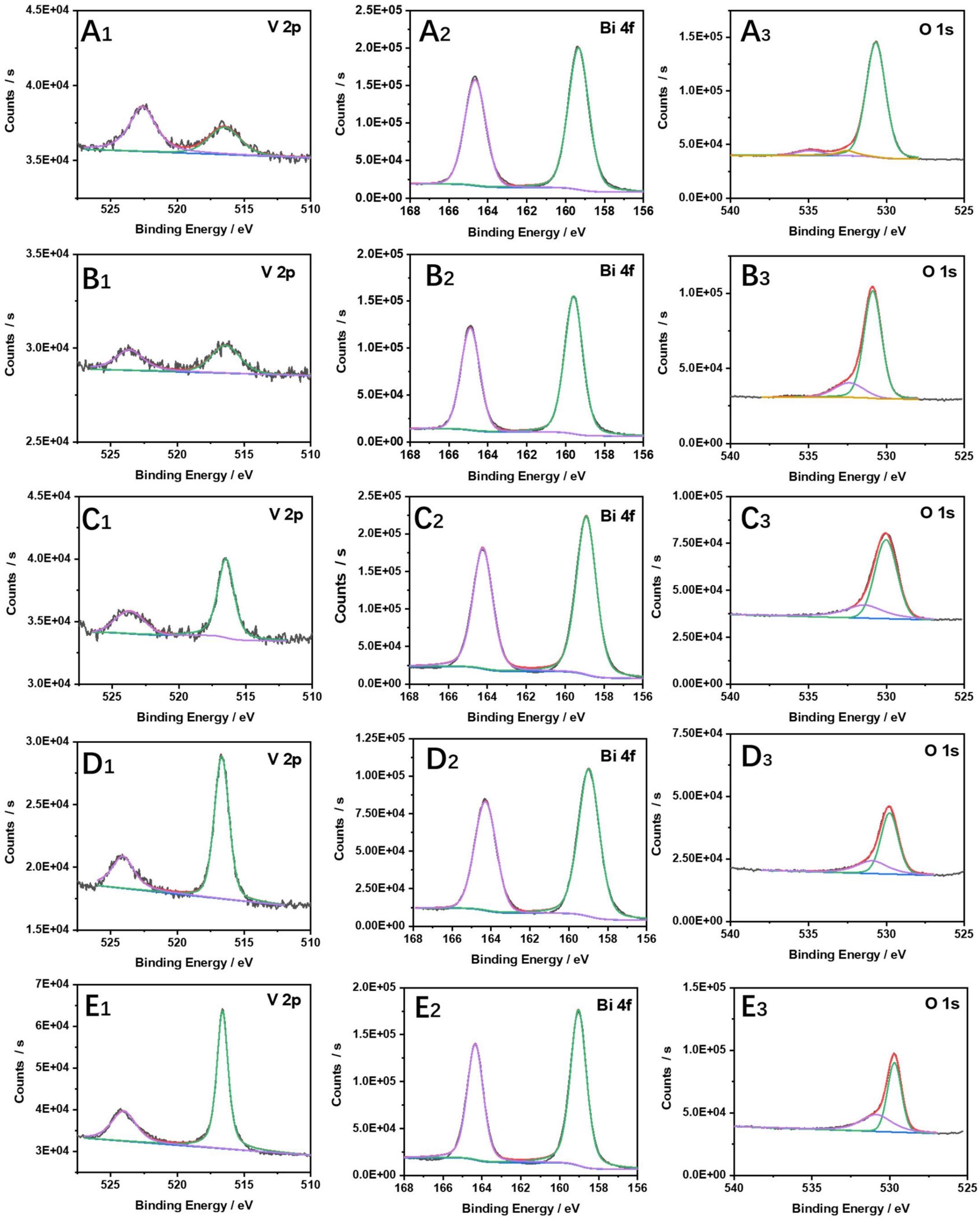

Figure S3. High-resolution XPS spectra of V 2p, Bi 4f and O 1s. (A) clavate (B) fusiform (C) flowered, (D) bulky and (E) particle $BiVO_4$.

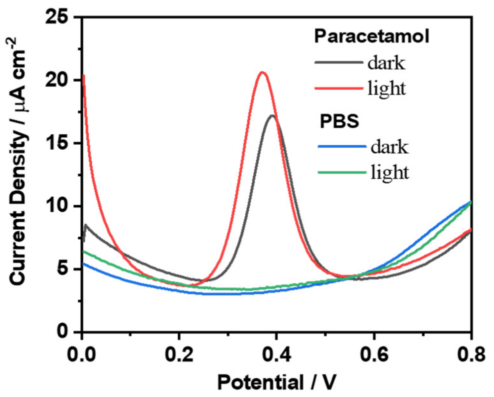

Figure S4. DPV curves of clavate $BiVO_4$ in 0.01 M PBS with and without 100 μM paracetamol. The DPV test was performance under dark condition (labeled as dark) and in illumination condition (labeled as light).

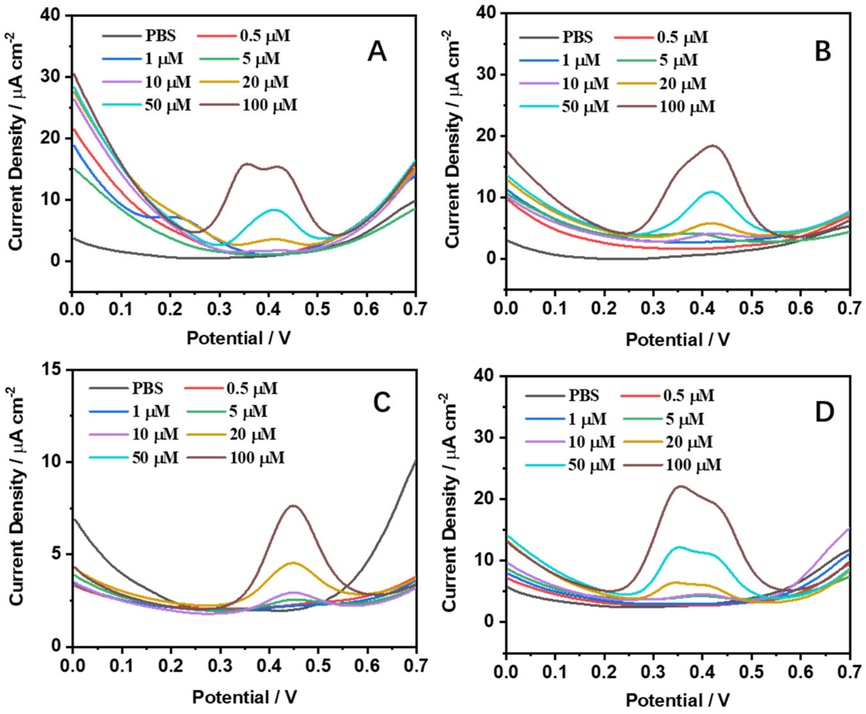

Figure S5. DPV curves at (A) fusiform (B) flowered, (C) bulky and (D) particle $BiVO_4$ in 0.01 M PBS with 0-100 μM paracetamol.

Table S1. Comparison between the present work and some reported sensors for paracetamol determination.

**Sensor**	Solution	Linear range	**LOD**	**Ref.**
MWCNTs/GO	0.05 M PBS (pH 7.0)	0.5-400 mM	47 nM	[1]
Au NPs	PBS (pH 7.4)	0.05-270 μM	14.6 nM	[2]
Au@graphene/ PEDOT	PBS (pH 7.0)	0.15-5.88 mM	41 nM	[3]
TiO_2-graphene	0.1 M PBS 0.2 (pH 7.0)	1-100 μM	0.21 μM	[4]
Co/MWCNTs	0.1 M PBS	5.2×10^{-3}-0.45 μM	1.0 nM	[5]
Pd/GO	0.1 M PBS (pH 6.8)	0.005-0.5 μM 0.5-80.0 μM	2.2 nM	[6]
PS-PNIPAm-PS/MWCNTs-COOH/N-GQDs	0.1 M PBS 0.2 (pH 7.0)	0.1-7.0 μM 7.0-103.0 μM	66 nM	[7]
Polymeric micelle	PBS solution (pH 7.0)	1×10^{-3} - 4 m M	0.33 uM	[8]
g-C_3N_4/PANI/ CdO	0.1 M PBS (pH 7.4)	0.1-790 mM	26 μM	[9]
Au–Ag	0.2 M PBS solution	0.01-0.1 mM	2.6 μM	[10]
NiAl LED	0.1 M acetate buffer	3-1500 μM	0.8 μM	[11]
$CoFe_2O_4$	0.1 M phosphate	3-200 μM	250 nM	[12]

	buffer (pH 6.0)			
NiO NPs	BR buffer (pH 3.0)	3.0-47.8 μM	0.12 μM	[13]
ZrO_2	PBS (pH 7.4)	10-60 μM	0.68 μM	[14]
Bi_2O_3	0.1 M KH_2PO_4	0.5-1500 μM	0.2 μM	[15]
Fe_2O_3 NPs	0.1 M PBS (pH 7.0)	2-170 μM	1.16 μM	[16]
Clavate-$BiVO_4$	0.01 M PBS (pH 7.4)	0.5-100 μM	0.2 μM	This work

LOD: limit of detection; PBS: phosphate buffer saline; BR buffer: Britton-Robison buffer; MWCNTs: multi-wall carbon nanotubes; GO: graphene oxide; PEDOT: Poly(3,4-ethylenedioxythiophene); PS-PNIPAm-PS: polymer poly(styrene-b-(N-isopropylacrylamide)-b-styrene); MWCNTs-COOH: carboxylated multi-walled carbon nanotubes; N-GQDs: amino-functionalized graphene quantum dots.

References

1. X. Kang, J. Wang, H. Wu, J. Liu, I. A. Aksay, Y. Lin, A graphene-based electrochemical sensor for sensitive detection of paracetamol, Talanta 2010, 3, 754-759.

2. N. F. Atta, A. Galal, S. M. Azab, Electrochemical determination of paracetamol using gold nanoparticles-application in tablets and human fluids, Int. J. Electrochem. Sci. 2011, 6 5082-5096.
3. M. Li, W. Wang, Z. Chen, Z. Song, X. Luo, Electrochemical determination of paracetamol based on Au@graphene core-shell nanoparticles doped conducting polymer PEDOT nanocomposite, Sens. Actuat. B-Chem. 2018, 260, 778-785.

4. Y. Fan, J. Liu, H. Lu, Q. Zhang, Electrochemical behavior and voltammetric determination of paracetamol on Nafion/TiO_2–

graphene modified glassy carbon electrode, Colloid. Surface. B 2011,85, 289-292.

5. A. Kutluay, M. Aslanoglu, An electrochemical sensor prepared by sonochemical one-pot synthesis of multi-walled carbon nanotube-supported cobalt nanoparticles for the simultaneous determination of paracetamol and dopamine, Anal. Chim. Acta 2014, 839, 59-66

6. J. Li, J. Liu, G. Tan, J. Jiang, S. Peng, M. Deng, D. Qian, Y. Feng, Y. Liu, High-sensitivity paracetamol sensor based on Pd/graphene oxide nanocomposite as an enhanced electrochemical sensing platform, Biosens. Bioelectron. 2014, 54, 468-475..

7. P. Zhao, M. Ni, C. Chen, Z. Zhou, X. Li, C. Li, Y. Xie, J. Fei, Stimuli-enabled switch-like paracetamol electrochemical sensor based on thermosensitive polymer and MWCNTs-GQDs composite nanomaterial, Nanoscale 2019, 11, 7394-7403.

8. J. Luo, C. Fan, X. Wang, R. Liu, X. Liu, A novel electrochemical sensor for paracetamol based on molecularly imprinted polymeric micelles, Sensor. Actuat. B-Chem 2013,188,909-916.

9. S. Bonyadi, K. Ghanbari, M. Ghiasi, All-electrochemical synthesis of a three-dimensional mesoporous polymeric g-C_3N_4/PANI/CdO nanocomposite and its application as a novel sensor for the simultaneous determination of epinephrine, paracetamol, mefenamic acid, and ciprofloxacin, New J. Chem. 2020, 44, 3412.

10. R. Wei, Biosynthesis of Au–Ag alloy nanoparticles for sensitive electrochemical determination of paracetamol, Int. J. Electrochem. Sci. 2017, 12, 9131-9140.

11. K. Asadpour-Zeynali, R. Amini, Nanostructured hexacyanoferrate intercalated Ni/Al layered double hydroxide modified electrode as a sensitive electrochemical sensor for paracetamol determination, Electroanalysis 2017, 29, 635-642.

12. Y. Kumar, P. Pramanik, D. K. Das, Electrochemical detection of paracetamol and dopamine molecules using nano-particles of cobalt ferrite and manganese ferrite modified with graphite, Heliyon, 2019, 5, e02031.

13. P. B. Deroco, F. C. Vicentini, O. Fatibello-Filho, An electrochemical sensor for the simultaneous determination of paracetamol and codeine uing a glassy carbon Electrode modified with nickel oxide Nanoparticles and carbon black, Electroanalysis 2015, 27, 2214-2220.

14. S. B. Matt, S. Raghavendra, M. Shivanna, M. Sidlinganahalli, D. M. Siddalingappa, Electrochemical detection of paracetamol by voltammetry techniques using pure zirconium oxide nanoparticle based modified carbon paste electrode, J. Inorg. Organomet. P. 2020, doi.org/10.1007/s10904-020-01743-y.

15. M. Zidan, T. W. Tee, A. H. Abdullah, Z. Zainal, G. J. Kheng, Electrochemical oxidation of paracetamol mediated by nanoparticles bismuth oxide modified glassy carbon Electrode, Int. J. Electrochem. Sci. 2011, 6, 279-288.

16. M. M. Vinay, Y. A. Nayaka, Iron oxide (Fe_2O_3) nanoparticles modified carbon paste electrode as an advanced material for electrochemical investigation of paracetamol and dopamine, J. Sci-Adv. Mater. Dev. 2019, 4, 442-450.
